# Keratitis due to *Chaetomium* sp.

**DOI:** 10.1155/2011/696145

**Published:** 2012-01-31

**Authors:** Jayaraman Kaliamurthy, Catti Munuswamy Kalavathy, Christadoss Arul Nelson Jesudasan, Philip A. Thomas

**Affiliations:** ^1^Institute of Ophthalmology, Joseph Eye Hospital, Tiruchirapalli 620 001, India; ^2^Department of Microbiology, Institute of Ophthalmology, Joseph Eye Hospital, Tiruchirapalli 620 001, India

## Abstract

*Aim*. To describe keratitis due to *Chaetomium* sp. occurring in a 65-year-old woman who presented with a corneal ulcer with hypopyon of the right eye with a history of trauma by vegetable matter. *Method*. Multiple scrapings were obtained from the ulcer. A lactophenol cotton blue wet mount and a Gram-stained smear of the scrapings were made. Scrapings were also inoculated onto various culture media. *Results*. Direct microscopy of corneal scrapings revealed moderate numbers of septate fungal hyphae. Greenish-yellow-coloured fungal colonies with aerial mycelium were observed in culture of the corneal scrapes. On the basis of colony characteristics and conidial structure, the fungal isolate was identified as *Chaetomium* sp. The patient was treated with topical natamycin (5%) hourly and cyclopentolate 1% drops 3 times a day. After 4 weeks of therapy, the hypopyon had disappeared, the epithelial defect had healed, and the stromal infiltration had almost completely resolved; the visual acuity of the eye improved from hand movements to (1/2)/60. *Conclusion*. Fungi of the genus *Chaetomium*, which are rare causes of human disease (systemic mycosis, endocarditis, subcutaneous lesions), may also cause ocular lesions.

## 1. Introduction

Mycotic keratitis is an important ophthalmological problem, particularly in agricultural communities, and more than 50 genera of fungi have been implicated in its aetiology [1]; most of these are saprobic organisms that cause opportunistic corneal infections in traumatized or immunologically compromised eyes. The genera of fungi most frequently implicated as causes of mycotic keratitis worldwide include *Aspergillus, Candida, Curvularia, Fusarium, *and *Penicillium.* In addition, certain other species of fungi may rarely cause keratitis; new infectious agents are continually appearing, around 20 species yearly. It is possible that many of the recently reported taxa have caused infections which previously passed unnoticed due to inadequate diagnostic expertise [2]. This paper describes a case of keratitis due to an infrequently reported fungal pathogen, *Chaetomium *sp*.*

## 2. Case Presentation

**Figure 1:**
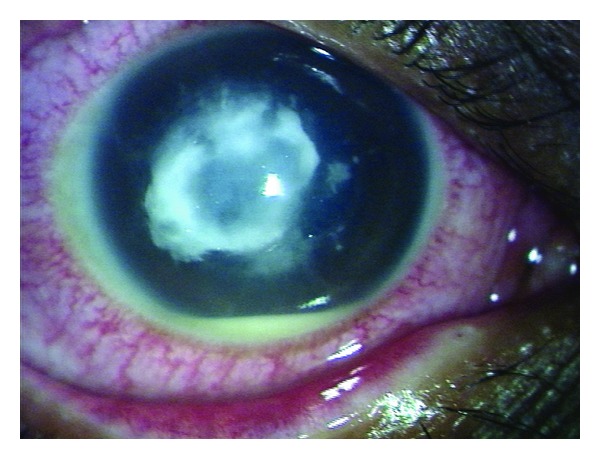


A 65-year-old man presented in February 2011 at the Joseph Eye Hospital, Tiruchirappalli, India. The patient complained of pain, redness, irritation, watering, and photophobia in the right eye (RE) following injury by vegetable matter (hay). At presentation, the patient was found to have a corneal ulcer (4 × 4 mm) with hypopyon (1 mm) in the RE (Figure 1); the visual acuity in this eye was “hand movements” (HM) only. The visual acuity in the patient's left eye was 6/18; no abnormality was detected in the left eye.

Under local anaesthesia (4% lignocaine), multiple scrapings were obtained from the base and edges of the corneal ulcer with a sterile blunt cataract knife. A lactophenol cotton blue (LPCB) wet mount and Gram-stained smear of the scrapings were prepared for direct microscopic examination. Scrapings were also inoculated onto plates of cystine tryptone agar and blood agar, which were incubated at 37°C, and Sabouraud glucose-neopeptone agar and broth (SDA, SDB), which were incubated at room temperature.

**Figure 2:**
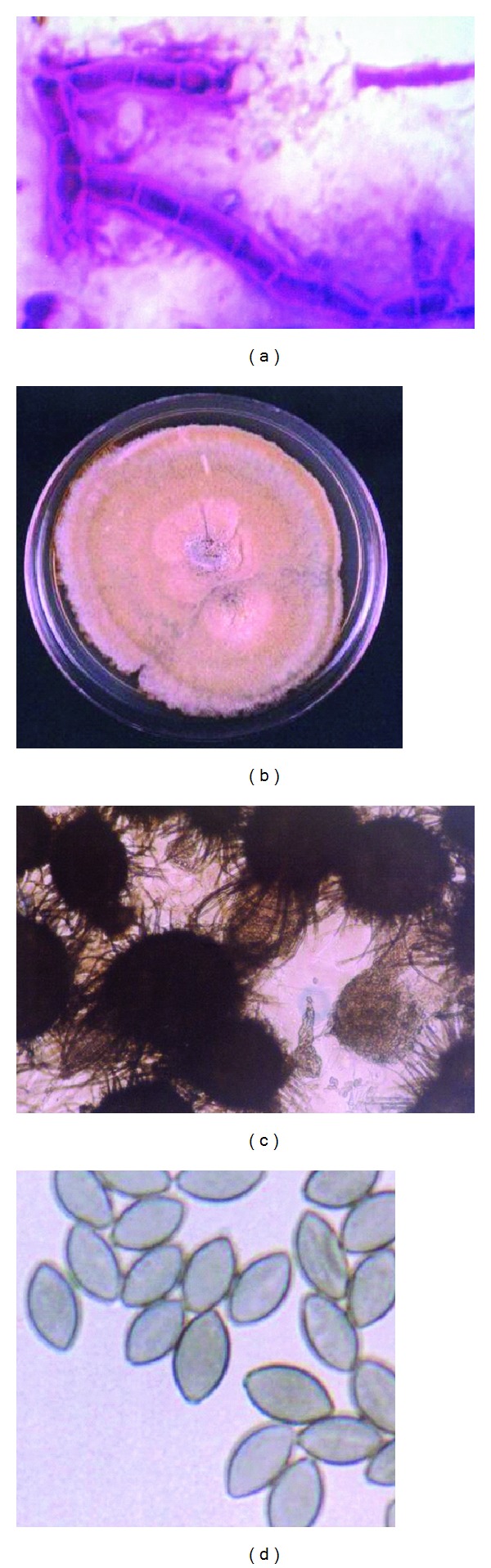


Direct microscopic examination of the LPCB wet mount as well as the Gram-stained smear revealed numerous septate fungal hyphae in the corneal scrape material (Figure 2(a)). Within five days of incubation, yellowish-green colonies with macroconidia were observed on all the plates. Bacteria were not isolated. Subcultures made on SDA (Figure 2(b)) and potato dextrose agar (PDA) showed fungal colonies with olivaceous aerial mycelium and yellow and green exudates. A cellophane tape mount prepared from the cultures (primary and subculture plates) revealed that the mycelium was composed of branching septate pigmented hyphae. Ascocarps (Figure 2(c)) were subglobose, pale brown, and ostiate with a cell wall consisting of interwoven hyphae. Ascocarps bore many thin-walled, hyphae-like appendages (setae). Ascospores (Figure 2(d)) were initially hyaline, becoming dark brown at maturity and were smooth, fusoidal, and rarely inequilateral. They had a single apical germ pore.

The patient was treated with topical natamycin (0.5%) hourly and cyclopentolate (1%) drops three times a day. After 4 weeks of therapy, the hypopyon had disappeared, the epithelial defect had healed, and the stromal infiltration had almost completely resolved; the visual acuity of the eye improved from HM to (1/2)/60. 

## 3. Discussion

Mycotic keratitis is an important ophthalmologic problem, particularly in rural workers. An increase in ocular infections produced by pathogenic or saprobic fungi has been documented [3, 4]. Many different fungal species have been reported as causative agents of keratitis, which is not surprising since they, as saprophytic inhabitants of soil, plants and trees, can be traumatically implanted into the cornea.

In this patient, there was a history of ocular trauma with vegetable matter (hay), which suggested the possibility of a fungal infection. Dwelling in an environment with dust pollution has been reported as a risk factor for mycotic keratitis [5, 6]. The seriousness of mycotic corneal infection should not be underestimated. If not promptly and properly treated, mycotic keratitis can lead to a marked diminution in vision, and, in severe cases, total blindness or even loss of the eye may result. In this patient, after 4 weeks of intensive medical therapy, the corneal lesion resolved and the visual acuity of the eye improved from HM to (1/2)/60.

Species of *Chaetomium* are widespread in soil and plant debris, where they are important agents of cellulose degradation. Species of *Chaetomium* are occasionally encountered as contaminants in clinical specimens; however, they sometimes pass unrecognized since they can present as non-sporulating moulds. Cases of confirmed human infection due to this fungus have been infrequently reported in the literature. However, *Chaetomium *species have been reported to cause systemic infections in patients with acute leukemia [7, 8], to cause onychomycosis [9] and to contaminate peritoneal dialysis fluid [10]. In addition, there have been reports of this fungus causing invasive infections in immunocompromised (due to renal transplantation) patients [11], brain abscess in drug addicts [12], and peritonitis in a patient on peritoneal dialysis [13]. Unfortunately, most of these patients died. A PubMed search using the key words “keratitis” and “*Chaetomium*” yielded two individual case reports of keratitis due to *Chaetomium* species [14, 15]. One of these patients was treated with topical natamycin and oral ketoconazole, whereupon the keratitis resolved completely [15]. The other patient received a variety of medications, starting with hourly natamycin (5%) eye drops, atropine (1%) eye drops twice daily, bacitracin/polymyxin ophthalmic ointment three times daily, and a two-week course of fluconazole (200 mg) orally every day; later, therapy with fluconazole (0.3%) eye drops every 2 h was initiated, which was used alternately with natamycin eye drops every 2 h. After 6 weeks of antifungal treatment, the fungus was eradicated from the lesion; however, a central stromal scar limited her best-corrected spectacle vision to 20/60 [14]. The patient described in the present paper was treated with natamycin 5% eye drops hourly, the ulcer resolved after 4 weeks, and the visual acuity of the eye improved from HM to (1/2)/60.

## 4. Conclusions

 Fungi of the genus *Chaetomium,* which are rare causes of human disease, such as systemic mycoses, endocarditis, and subcutaneous lesions, may also cause corneal lesions.

## References

[1] Thomas PA (2003). Current perspectives on ophthalmic mycoses. *Clinical Microbiology Reviews*.

[2] Guarro J, Gené J, Stchigel AM (1999). Developments in fungal taxonomy. *Clinical Microbiology Reviews*.

[3] Kaliamurthy J, Kalavathy CM, Ramalingam MDK, Prasanth DA, Jesudasan CAN, Thomas PA (2004). Keratitis due to a coelomycetous fungus: case reports and review of the literature. *Cornea*.

[4] Kaliamurthy J, Jesudasan CAN, Prasanth DA, Thomas PA (2006). Keratitis due to *Cylindrocarpon lichenicola*. *Journal of Postgraduate Medicine*.

[5] Leck AK, Thomas PA, Hagan M (2002). Aetiology of suppurative corneal ulcers in Ghana and South India, and epidemiology of fungal keratitis. *British Journal of Ophthalmology*.

[6] Thomas PA, Geraldine P (2007). Infectious keratitis. *Current Opinion in Infectious Diseases*.

[7] Barale T, Fumey MH, Rebous G, Mallea M (1990). A case of *Chaetomium sp.* septicemia in a leukemic child associated with a bone-marrow transplant. *Bulletin de Societie Francaise Mycologiel Medicale*.

[8] Hoppin EC, McCoy EL, Rinaldi MG (1983). Opportunistic mycotic infection caused by *Chaetomium* in a patient with acute leukemia. *Cancer*.

[9] Latha R, Sasikala R, Muruganandam N, Shiva Prakash MR (2010). Onychomycosis due to ascomycete *Chaetomium globosum*: a case report. *Indian Journal of Pathology and Microbiology*.

[10] Febré N, Silva V, Medeiros EAS (1999). Contamination of peritoneal dialysis fluid by filamentous fungi. *Revista Iberoamericana de Micologia*.

[11] Anandi V, John TJ, Walter A (1989). Cerebral phaeohyphomycosis caused by *Chaetomium globosum* in a renal transplant recipient. *Journal of Clinical Microbiology*.

[12] Abbott SP, Sigler L, McAleer R, Mcgough DA, Rinaldi MG, Mizell G (1995). Fatal cerebral mycoses caused by the ascomycete *Chaetomium strumarium*. *Journal of Clinical Microbiology*.

[13] Barthez JP, Pierre D, de Bievre C, Arbeille M (1984). Peritonite a *Chaetomium globosum* chez un insuffisant renal traite part D.P.C.A. *Bulletin de Societie Francaise Mycologiel Medicale*.

[14] Mootha VV, Shahinpoor P, Sutton DA, Xin L, Najafzadeh MJ, de Hoog GS Identification problems with sterile fungi, illustrated by a keratitis due to a non-sporulating *Chaetomium*-like species.

[15] Balne PK, Nalamada S, Kodiganti M, Taneja M (2012). Fungal keratitis caused by *Chaetomium atrobrunneum*. *Cornea*.

